# Integrating spatial and ecological information into comprehensive biodiversity monitoring on agricultural land

**DOI:** 10.1007/s10661-023-11618-7

**Published:** 2023-09-07

**Authors:** Klaus Thomas Ecker, Eliane Seraina Meier, Yves Tillé

**Affiliations:** 1 Biodiversity and Conservation Biology Research, Swiss Federal Institute for Forest, Snow and Landscape Research WSL, Birmensdorf, Switzerland; 2 Agroecology and Environment, Agroscope, Zurich, Switzerland; 3 Institute of Statistics, University of Neuchâtel, Neuchâtel, Switzerland

**Keywords:** Balanced sampling, Habitat, Multi-stage, Vegetation, Geographic spreading, Unequal probability

## Abstract

Biodiversity loss on agricultural land is a major concern. Comprehensive monitoring is needed to quantify the ongoing changes and assess the effectiveness of agri-environmental measures. However, current approaches to monitoring biodiversity on agricultural land are limited in their ability to capture the complex pattern of species and habitats. Using a real-world example of plant and habitat monitoring on Swiss agricultural land, we show how meaningful and efficient sampling can be achieved at the relevant scales. The multi-stage sampling design of this approach uses unequal probability sampling in combination with intermediate small-scale habitat sampling to ensure broad representation of regions, landscape types, and plant species. To achieve broad coverage of temporary agri-environmental measures, the baseline survey on permanent plots is complemented by dynamic sampling of these specific areas. Sampling efficiency and practicality are ensured at all stages of sampling through modern sampling techniques, such as unequal probability sampling with fixed sample size, self-weighting, spatial spreading, balancing on additional information, and stratified balancing. In this way, the samples are well distributed across ecological and geographic space. Despite the high complexity of the sampling design, simple estimators are provided. The effects of stratified balancing and clustering of samples are demonstrated in Monte Carlo simulations using modelled habitat data. A power analysis based on actual survey data is also presented. Overall, the study could serve as a useful example for improving future biodiversity monitoring networks on agricultural land at multiple scales.

**Supplementary Information:**

The online version contains supplementary material available at 10.1007/s10661-023-11618-7.

## Introduction

Agriculture is one of the main causes of the massive loss of biodiversity in recent decades. In Europe, historically established biodiversity on agricultural land has declined dramatically since the beginning of the twentieth century with the progressive intensification of farming (Allan et al., 2014; Richner et al., 2015). In Switzerland, for example, around 95% of seminatural grassland has disappeared (Lachat et al., 2010). Further changes are expected in the future due to climate change, demographic shifts, and changing productivity values (Debonne et al., 2022). The negative impacts of biodiversity loss on important ecosystem services, such as pollination, pest control, and soil conservation, are well known (Allan et al., 2015; Soliveres et al., 2016). Agri-environmental measures have therefore been introduced to enhance existing biodiversity by paying farmers for appropriate management. This public expenditure is significant, and the effectiveness of agri-environmental measures therefore needs to be carefully monitored (Sutcliffe et al., 2015; Batáry et al., 2015; Herzog & Franklin, 2016).

Comprehensive biodiversity monitoring requires the collection of data on habitats and species in a representative sample of the target area. For the selection of the sample, a probability sample is essential in order to draw valid statistical conclusions about the target area based on probability theory (Tillé, 2020; Lohr, 2022). Current approaches to biodiversity monitoring on agricultural land rely on equal probability sampling, such as simple random sampling or spatial grid sampling, to produce reliable national statistics (e.g. Biodiversity Monitoring Switzerland (BDM Coordination Office, 2014)). In doing so, most large-scale biodiversity monitoring approaches have adopted a stratified approach (Herzog & Franklin, 2016) to ensure better representation of smaller regions and rare landscape types (Metzger et al., 2013) and use two-stage sampling to cover multiple units per selected site (Carey et al., 2008; Pascher et al., 2011). Spatial concentration of the survey effort reduces travel costs, which allows a larger sample size and thus broader sample coverage. Two-stage approaches are also targeted for simultaneous monitoring of biodiversity at local and landscape scales. The inclusion of both scales is important, for example, to study the effects of land-use intensification on biotic homogenisation (Gossner et al., 2016). The landscape scale as such is particularly important for mobile taxa at higher trophic levels; for example, butterflies and birds often have different areas for foraging and nesting, so that the effects of management may be less evident at the local scale than at the landscape scale (Meier et al., 2022). Unfortunately, the biodiversity monitoring approaches described so far are not very effective at monitoring biodiversity on small-scale agricultural land, where a few species and habitats are predominant and widespread, while most species and habitats tend to be rare or spatially clumped. In this setting, most elements of interest are not adequately sampled at the local scale. If, on the other hand, additional small-scale information, e.g. from habitat mapping and remote sensing, is obtained, a stratified approach can be adopted in the second stage of sampling to cover a broader range of local biodiversity (Bunce et al., 2011).

In addition to broad sample coverage, for estimation efficiency reasons, samples should be well spread across the variables known to influence the variables we are interested in estimating (i.e. indicators) (Tillé, 2011). Specifically, since the distribution of habitats and species is influenced by environmental variables, such as climate, topography, and soil properties (Guisan et al., 2017), biodiversity samples should be well spread across these variables. Spreading across geographic space (Grafström et al., 2012) is additionally important, as species and habitats that are close to each other may not be independent of each other, due to common management, incomplete dispersal processes, and biotic interactions through species competition (Legendre & Legendre, 1998).

There is currently no large-scale monitoring approach using probability sampling that simultaneously covers a broad range of local, landscape, and regional biodiversity on agricultural land, features an optimal distribution of the sampling units across ecological and geographic space, and yet is suitable for operational field campaigns. Furthermore, there is no existing probability sampling approach that includes a nationwide assessment of agri-environmental measures (Herzog & Franklin, 2016) to evaluate their contribution to biodiversity on agricultural land and to adjust them as needed in a timely manner.

Here, we present a long-term monitoring approach for species, habitats, and structures on the agricultural land of Switzerland (‘ALL-EMA’; http://www.allema.ch). ALL-EMA takes into account all the above requirements by using modern sampling techniques, such as unequal probability sampling with fixed sample sizes, balancing on auxiliary information (Deville & Tillé, 2004), stratified balancing (Chauvet, 2009), spatial spreading (Grafström & Tillé, 2013), two-phase sampling (see Särndal et al., 1992, pp. 343–385), and self-weighting (see Särndal et al., 1992, pp. 132–154). Unequal probability sampling allows sampling rates to be adjusted in a way similar to stratification, but without the need to introduce artificial boundaries. In combination with spatial spreading and balancing, it allows additional spreading in geographic and environmental space. Meanwhile, the stratified balancing method, which is balancing on categorical variables, retains control over the sample sizes within strata. These techniques have already been used in sampling designs for forest inventories (Vallée et al., 2015), conservation monitoring (Tillé & Ecker, 2014), land-use and land-cover estimation (Fattorini et al., 2015), soil mapping (Brus, 2019), and population surveys (Fattorini & Ferretti, 2020). However, these studies have focused on landscapes with biodiversity patterns less complex than those on agricultural land or have pursued objectives other than biodiversity assessment.

The aim of the ALL-EMA monitoring approach is to represent a wide variety of plant species, habitat types, and agri-environmental measures and to efficiently estimate predefined biodiversity indicators for a range of target groups and at different scales. The practical example of Switzerland is well suited to assess the effectiveness of agri-environmental measures (e.g. ecological focus areas (EFAs)) on a large scale, as these measures are linked to subsidies through a cross-compliance mechanism, which means that almost all farmers implement such measures on their farms. However, as this is a real-world example, additional constraints have to be taken into account, e.g. an existing national but landscape-level survey of plants, butterflies, and breeding birds in grid squares of 1 km^2^ (BDM-Z7 sample) (BDM Coordination Office, 2014) has to be integrated. The survey is therefore conducted on a subset of the BDM-Z7 grid squares and follows its rotational survey schedule. Another challenge of the ALL-EMA monitoring design is to divide the sampling effort between the squares of the BDM-Z7 sample, the target regions, and the habitat and species survey and to cover a wide variety of EFAs.

The ALL-EMA sampling scheme that was ultimately implemented defines two types of surveys: a baseline survey of permanent plots to record biologically relevant structures, habitat types, and plant species on the whole agricultural land, and an additional survey of non-permanent plots to record the same information from the EFAs that may change location over time. The sampling of both surveys is organised into three stages. In the first stage, a targeted sample of squares is selected from the initial BDM-Z7 squares. In a second stage, a spatial grid of nested circular plots of 10 m$^2$ and 200 m$^2$ is defined within the first-stage sampling units for the baseline survey of habitat types, structures, and neophytes on the agricultural land in a square. Next, a targeted subsample of the 10 m$^2$ plots is selected for the baseline survey of plant species composition. The sampling design of the baseline survey thus includes two-phase sampling to generate *a priori* missing information on habitats for a broader survey of the plant species. For the survey of the dynamic EFAs, two additional stages of sampling are used within the same first stage selection of BDM-Z7 squares. First, one plot is randomly placed within each EFA of a square. A targeted subsample of these intermediate plots is then selected for the concurrent survey of habitat types and plant species. Sampling in the three-stage ALL-EMA sampling procedure is carried out using modern techniques. In both survey types, unequal probability sampling is used in the first and third stage of sampling to achieve a broader representation of target regions, plant species, or EFA categories. In each case, unequal probability sampling is combined with balancing on additional information, stratified balancing, geographic spreading, and self-weighting to achieve optimal efficiency.

In the following sections, we describe how the ALL-EMA sampling design was developed and the expected efficiency of some of the sampling procedures. We first present the sample population and the target parameters, including the underlying field data, in the ‘‘Population and target parameters’’ section. In the ‘‘Organisation of the ALL-EMA survey’’ section, we give an overview of the organisation of the ALL-EMA design, presenting the survey samples and notation, the main ideas and stages of sampling, and the rationale for sample sizes. In the ‘‘Sampling design in full detail’’ section, we describe the sampling procedures in full detail. We then present simple point and variance estimators for plot-level estimation in the ‘‘Estimators’’ section. We assess the estimation efficiency of the sampling procedure in the ‘‘Sample efficiency assessment’’ section, using Monte Carlo simulations based on modelled data and conducting power analyses based on recent survey data. Finally, we provide a brief conclusion on the ALL-EMA sampling design in the ‘‘Conclusions’’ section. Further information on the full sampling design of the EFA survey, the temporal organisation of the survey, the characteristics of the final square sample, additional examples of estimation, and general comments on estimation in space and time, as well as power analyses based on simulations, can be found in the Supplementary Information.

## Population and target parameters

### Population and sampling frame

ALL-EMA aims to assess the entire agricultural area of Switzerland, which makes up 38% of the national surface area of 41,285 km$^2$. Grassland is the predominant land-cover type, covering 79% of this agricultural land. The remainder is occupied by arable land, vineyards, orchards, and special cultures (Herzog et al., 2017). For practical reasons, the target population is restricted to accessible areas. Therefore, the aim is to cover the full area of Switzerland, excluding forests, settlements, and lakes, as well as unproductive and inaccessible areas. However, the extent of the target area is not known exactly. In the sampling frame, a preliminary delineation of the agricultural land from a GIS database is used to represent the survey area. This delineation is checked and updated in the field, and this process is repeated for each survey run.

The agricultural land is further divided into agricultural production zones (ERZOs) and biogeographic regions (UZL-HRs) (Walter et al., 2013). These areas are the relevant units for evaluating the EFAs. As EFAs can emerge and disappear over time, they are included in the sampling frame as annual delineations.

**Fig. 1:**
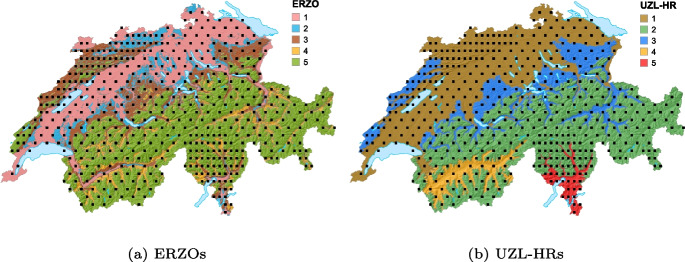
Systematic distribution of the initial grid squares (1 km^2^) of the existing landscape-level survey of plants, butterflies, and breeding birds (BDM-Z7), with double grid density in some areas in Switzerland. Two types of geographic stratifications are used in the sampling design: **a** agricultural production zones (ERZOs) and **b** biogeographic regions (UZL-HRs). ERZOs: 1 = valley zone, 2 = hill zone, 3 = lower mountain zones I and II, 4 = upper mountain zones III and IV, 5 = summering zone. UZL-HRs: 1 = Central Plateau and low areas of the Jura mountains, 2 = Alps, 3 = high western Jura mountains and low areas in the Alps, 4 = low areas in the canton of Valais, 5 = southern Alpine fringe

The ALL-EMA sampling is restricted to the BDM-Z7 sampling units, a grid of sample squares of 1 km$^2$ (BDM Coordination Office, 2014). The grid density is doubled in the Southern Alps and Jura regions (Fig. 1). The raw grid has 509 squares. In the BDM-Z7 survey, 35 squares are excluded because they are covered completely by glaciers, lakes, or inaccessible terrain. A further 19 squares do not contain any accessible agricultural land. Thus, the sampling frame of the ALL-EMA survey encompasses 455 BDM-Z7 squares.

### Target groups and indicators

The ALL-EMA survey covers the entire agricultural area of Switzerland, but has various predefined geographical and thematic subgroups for separate evaluation. Of particular interest are (a) the five ERZOs, i.e. valley zone, hill zone, lower mountain zones I and II, upper mountain zones III and IV, and summering zone (Fig. 1a); (b) 23 types of EFA; and (c) habitat types, partitioned into 91 categories.

Target groups of secondary importance are (a) the five biogeographic regions (UZL-HRs), namely the Central Plateau and low areas of the Jura mountains, the Alps (including its low areas) plus the high western Jura mountains, low areas in the canton of Valais, and the southern Alpine fringe (Fig. 1b), and (b) the individual sampling squares.

The biodiversity of the agricultural land is assessed using a predefined list of indicators that can be divided into four main thematic groups: (a) the diversity of species, habitats, and structures; (b) the area share of species, habitats, and structures; (c) the length of linear structures including water bodies; and (d) mean trait/indicator values of the species (e.g. mean nutrient value of the plant species according to Landolt et al., 2010). Each thematic group is represented by several indicators derived from the field data (Meier et al., 2021). Common methods are used to quantify the diversity measures beyond the plot scale (e.g. $\gamma$-diversity; Magurran & McGill, 2011).

### Field data

**Fig. 2:**
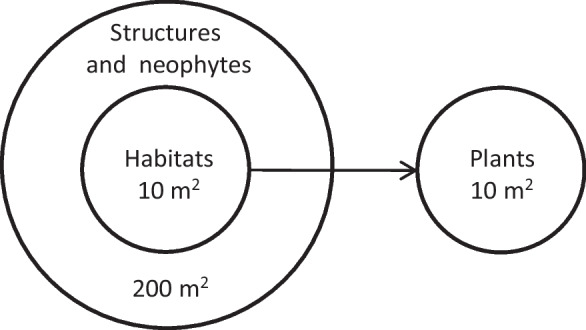
Geometric representation of a nested circular plot in the ALL-EMA survey. Information on structures and neophytes is collected within a 200 m$^2$ circle. Habitat type and plant species composition are recorded within a 10 m$^2$ circle

To calculate the indicators, in situ information on biologically relevant structures, habitat types, and plant species is collected in nested circular plots (Fig. 2). Habitat types are usually classified in terrestrial habitats based on phytosociological classification (mainly vascular plants). This is also the basis for the hierarchical classification adapted to Swiss habitat types by Delarze et al. (2015). In the case of ALL-EMA, the habitats are recorded at the third level of detail (i.e. TypoCH) by using a dichotomous field protocol based on the occurrence of characteristic plant species.

## Organisation of the ALL-EMA survey

### Survey samples and notation

**Fig. 3:**
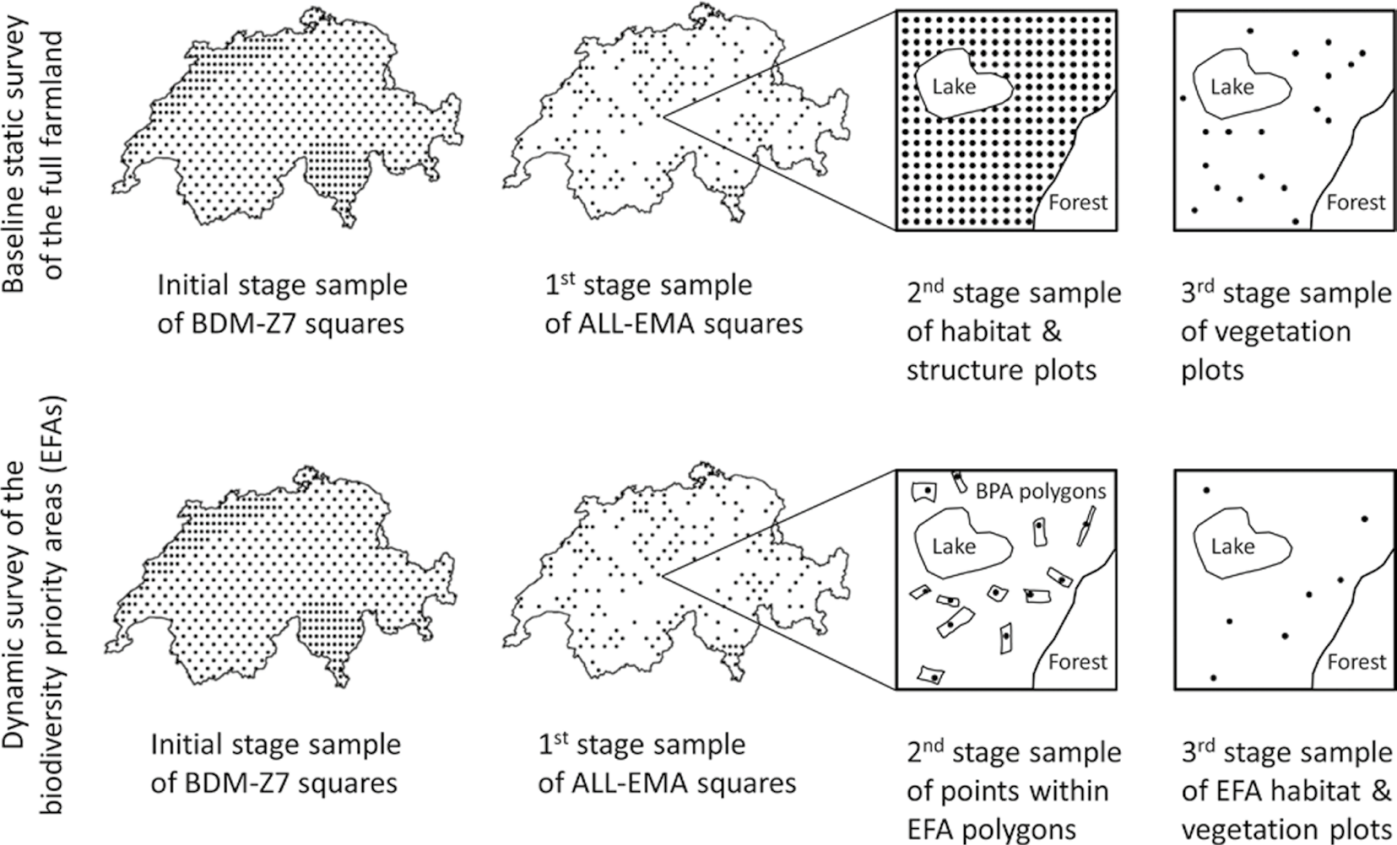
Overview of the multi-stage sampling design of ALL-EMA for the baseline static survey of the full agricultural land in Switzerland and the dynamic survey of the temporary ecological focus areas (EFAs). In the first stage of sampling, a targeted sample of squares is selected from the initial grid squares of the Swiss landscape-level survey of plants, butterflies, and breeding birds (BDM-Z7). The second stage of sampling is a spatial grid sample of circular plots within the first-stage selection of squares for the survey of habitat types, structures, and neophytes in the agricultural land of a square. In the third stage of sampling, a targeted subsample of the habitat plots is selected for the survey of plant species composition. The survey of the dynamic EFAs is based on two extra stages of sampling within the same first stage selection of BDM-Z7 squares. After placing one random point within each EFA polygon of a square, a targeted subsample of these intermediate points is selected for the concurrent survey of habitat types and plant species. The sampling of the EFAs is repeated for each survey run. In both survey types, unequal probability sampling is combined with balancing on additional information, stratified balancing, geographic spreading, and self-weighting to achieve optimal estimation efficiency and controlled sample sizes

The complex sampling design of ALL-EMA defines two samples of permanent plots for the baseline and static survey of habitats and plant species in the entire agricultural area of Switzerland (Fig. 3, top). To ensure a sufficient number of plots in the EFAs and to cope with their dynamic character, an additional sample of non-permanent plots is drawn per survey run from this target group (Fig. 3, bottom). The notation for the different samples and the respective inclusion probabilities are given in Tables 1 and 2.

### Rationale of the sampling design

For both types of surveys, multi-stage and unequal probability sampling are used to distribute the workload across the initial BDM-Z7 sampling squares and to sample the target groups (i.e. regions/zones, habitats, and EFAs) according to the predefined interests. The sampling concept of the baseline survey involves a two-stage approach for this purpose, in order to first collect dense but cost-effective information on habitat types, which is then used for targeted sampling of plant species. The redundant information on plant species from the large regions and the predominant habitat types and EFAs is thus reduced. The selection of the BDM-Z7 squares is also proportional to the area of the agricultural land within a square in order to later obtain an efficient self-weighted (see Särndal et al., 1992, pp. 132–154) vegetation sample of fixed sample size within the squares. To match the annual rotation plan of the BDM-Z7 survey, the selection of the BDM-Z7 squares is temporally stratified with a fixed sample size per survey year. The annual subsamples are spread in geographic space and balanced on auxiliary variables. Balancing improves estimation efficiency if the variable of interest is linearly dependent on the auxiliary variables. Spreading has the same effect when the variable of interest has a geographic structure or trend. The fixed sample sizes are convenient for the organisation of the field work (see the ‘‘Sampling design in full detail’’ section for details of the full sampling design).

### Stages of sampling

The sampling for the baseline survey is organised into three stages additional to the BDM-Z7 sample (Fig. 3, top). In the first stage, a sample $S_1$ of $m=170$ sampling units is selected from the initial sample $S_0$ of $M=455$ BDM-Z7 squares. In the second stage, a large sample $S_2$ of circular plots is drawn within the first-stage sampling units $S_{1}$. Plot centres are located on a systematic grid, with a grid spacing of 50 m. Data about habitat type and biologically relevant structures and neophytes are collected with sample $S_2$ in nested circular plots of 10 m$^2$ and 200 m$^2$. In the third stage, a subsample $S_3$ is selected from $S_2$ for the survey of plant species composition in plots of 10 m$^2$ (Fig. 2). Listing all the plants occurring within a plot is time-consuming and cost-intensive, and the affordable sample size is thus much smaller.

The sampling design for the additional EFA sample is again organised into three stages (Fig. 3, bottom). The first-stage sample $S_{1_{E}}$ is largely identical to the first-stage sample $S_{1}$ of BDM-Z7 squares in the baseline survey and only differs in the area extent of the sampling squares. In the second stage of sampling, one nested circular plot is randomly located in each EFA polygon to form the intermediate sample $S_{2_{E}}$. In the third stage, a subsample $S_{3_{E}}$ is drawn from $S_{2_{E}}$ for the survey of structures, neophytes, habitats, and plant species on the same set of EFA plots. The respective plot sizes are the same as those in the baseline survey (Fig. 2).

**Table 1:** The three stages of the baseline sampling design for the repeated survey of habitats and species in the entire agricultural area based on permanent plots. A sample $S_1$ of squares is selected from the initial BDM-Z7 sample $S_0$. Plots are then selected in the $S_1$ squares for the habitat sample $S_2$. Finally, a subsample of the plots in the $S_2$ habitat sample is selected for the vegetation sample $S_3$. For simplicity, the sample for recording structures and neophytes is not listed in the table. This sample differs from $S_2$ only in the larger plot size of 200 m$^2$ and thus inclusion probabilities

Name of	Notation	Sample	Area of	Conditional	Total
the sample		size	sample unit	inclusion probability	inclusion probability
Baseline initial BDM-Z7	$S_0$	$M=455$	0.9025 km$^2$ (squares)	$\pi _{0,i}$	$\pi _{0,i}$
Baseline first stage	$S_1$	$m=170$	0.9025 km$^2$ (squares)	$\pi _{1,i\mid i\in S_0}$	$\pi _{1,i} = \pi _{0,i} \;\pi _{1,i\mid i\in S_0}$
Baseline second stage (habitat sample)	$S_2$	$n=\sum _{i\in S_1} n_i \approx 36,000$	10 m$^2$ (circular plots)	$\pi _{2,j\mid i\in S_1}$	$\pi _{H,j}= \pi _{1,i}\; \pi _{2,j\mid i\in S_1}$
Baseline third stage (vegetation sample)	$S_3$	$v=\sum _{i\in S_1} v_i \approx 3,200$	10 m$^2$ (circular plots)	$\pi _{3,j\mid j\in S_2}$	$\pi _{V,j}=\pi _{H,j}\pi _{3,j\mid j\in S_2}$

**Table 2:** The three stages of the EFA sampling design for the additional repeated survey of habitats and species in the EFA polygons based on dynamic sampling units. A sample $S_{1_E}$ of squares is selected from the initial sampling frame $S_{0_E}$. The selection corresponds to the sample squares $S_1$ of the baseline survey. The squares only differ in area. One plot is then randomly placed in each EFA polygon belonging to the selected squares ($S_{2_E}$ sample). Finally, a subsample of the plot sample $S_{2_E}$ is drawn for a joint sampling of habitats and vegetation $S_{3_E}$. For simplicity, the sample for recording structures and neophytes is not listed in the table. This sample differs from $S_{3_E}$ only in the larger plot size of 200 m$^2$ and thus inclusion probabilities

Name of	Notation	Sample	Area of	Conditional	Total
the sample		size	sample unit	inclusion probability	inclusion probability
EFA initial BDM-Z7	$S_{0_E}$	$M=455$	1 km$^2$ (squares)	$\pi _{0_E,i}$	$\pi _{0_E,i}$
EFA first stage	$S_{1_E}$	$m=170$	1 km$^2$ (squares)	$\pi _{1_E,i\mid i\in S_{0_E}} = \pi _{1,i\mid i\in S_{0}}$	$\pi _{1_E,i}=\pi _{0_E,i}\pi _{{1_E},i\mid i\in S_{0_E}}$
EFA second stage	$S_{2_E}$	$p=\sum _{i\in S_{1_E}} p_i$	10 m$^2$ (circular plots)	$\pi _{2_E,j\mid i\in S_{1_E}}$	$\pi _{2_E,j}=\pi _{1_E,i}\pi _{2_E,j\mid i\in S_{1_E}}$
EFA third stage (joint sample)	$S_{3_E}$	$e=\sum _{i\in S_{1_E}} e_i \approx 2,400$	10 m$^2$ (circular plots)	$\pi _{3_E,j\mid j\in S_{2_E}}$	$\pi _{HV_E,j}=\pi _{2_E,j}\pi _{3_E,j\mid j\in S_{2_E}}$

### Rationale for sample sizes

The sample sizes given in Tables 1 and 2 are based on several considerations. The size $M=455$ of the $S_0$ sample of squares was predetermined by the existing sample of BDM-Z7 squares including accessible agricultural land.

The grid spacing for habitat sampling in the second stage of the baseline survey was also predefined to be 50 m. This grid resolution was found to optimally reflect the short-range variability in the Swiss agricultural land. With this spacing, the plot locations also match the observation points of the Swiss land-cover statistic (Arealstatistik) (GEOSTAT, 1997), a repeated survey over all of Switzerland based on aerial photo interpretation on a systematic 100 m $\times$ 100 m grid.

The high degree of sample clustering was intended to allow inferences at the square (i.e. landscape) level, but also proved to be a good trade-off with plot-level estimation, as demonstrated by a Monte Carlo simulation study investigating the impact of sample clustering on global habitat estimation (see “Effect of sample clustering”). The field budget for the baseline survey was to be shared equally between the habitat survey and the vegetation survey. Later, an additional budget was drawn up for the survey of the EFAs.

## Sampling design in full detail

### Initial-stage sample of the baseline BDM-Z7 squares

The initial BDM-Z7 sample of squares $S_0$ is the virtual sampling frame of the ALL-EMA survey. The probability that a square is included in the ALL-EMA sample frame is determined by the grid spacing of the BDM-Z7 sample, which varies according to biogeographic strata. While the standard spacing between squares in the BDM-Z7 sample is 12 km in the east–west direction and 8 km in the north–south direction, this grid density is doubled in the Southern Alps and in the Jura region. Thus, the regular grid densities are one BDM-Z7 square per 96 km^2^ and one BDM-Z7 square per 48 km^2^, respectively (Fig. 1).

In the BDM-Z7 sample, the squares are 1 km^2^, but because only $19\times 19 = 361$ habitat plots in a square grid of 50 m $\times$ 50 m are laid out in the ALL-EMA survey (Fig. 2), the actual size of a BDM-Z7 square in the baseline survey of ALL-EMA is 950 m $\times$ 950 m. The resulting inclusion probabilities of the BDM-Z7 squares are set to $\pi _{0}=0.9025/48= 0.01880208$ in the Southern Alps and in the Jura region and to $\pi _{0}=0.9025/96= 0.00940104$ otherwise.

### First-stage sample of the baseline BDM-Z7 squares

#### General principles

In the first stage of sampling, the initial BDM-Z7 squares are sampled with unequal probabilities to achieve higher sampling rates in the small target zones/regions and to favour the selection of squares with a larger share of accessible agricultural land, which in turn benefits a self-weighted vegetation sample with fixed sample sizes in the third stage of sampling. The first-stage sampling is conducted such that a balanced sample of maximum spatial spreading and equal sample size is achieved in the five annual panels of the BDM-Z7 survey.

The development of this first stage sample can be described using the following notation:

- *A* is the surface area of the accessible agricultural land of Switzerland, the target population of the ALL-EMA survey
- $i = 1, \ldots , M$ denotes the initial BDM-Z7 squares ($M = 455$)
- $A_{i}$ is the surface area of the accessible agricultural land within square *i*
- $n_i$ is the number of habitat plot centres located on accessible agricultural land in square *i*
- $j = 1, \ldots , n_i$ denotes the habitat plots in square *i*

#### Index of interest

In ALL-EMA, the aim is to form a reasonably large sample of BDM-Z7 squares in all ERZOs and UZL-HRs. To guide the first-stage unequal probability selection of squares from the initial BDM-Z7 squares, it is necessary to define an index of interest $I_i > 0$ on all BDM-Z7 squares, which fulfils the constraints:

1 $$\sum _{i \in U_g} \frac{A_{i}}{A} I_i = q_g,\;\text{for all strata}\;U_1,\dots ,U_g,\dots , U_G$$

2 $$\sum _{i\in V_h} \frac{A_{i}}{A} I_i = p_h,\;\text{for all strata}\;V_1,\dots ,V_h,\dots ,V_H$$

where the summation is over all *M* squares of the initial BDM-Z7 sample, with the centre point in stratum $U_g$ or $V_h$. $U_1,\dots ,U_g,\dots ,U_G$ denote the ERZOs and $V_1,\dots ,V_h,\dots ,V_H$ the UZL-HRs, and $q_{g}$ and $p_{h}$ are the proportions of the first-stage sample of squares that should fall into the respective zones and regions. They are defined as:

$$\begin{aligned} q_g = \frac{ A_{U_{g}}^\alpha }{\sum _{g=1}^G A_{U_{g}}^\alpha } \,\,\quad \text {and}\quad p_h = \frac{ A_{V_{h}}^\alpha }{\sum _{h=1}^H A_{V_{h}}^\alpha } \end{aligned}$$

where $A_{U_{g}}$ and $A_{V_{h}}$ denote the surface area of UZL-HR $U_{g}$ and of ERZO $V_{h}$ in the target population *A*. $\alpha$ is a parameter known from the power allocation of sampling units to the sampling strata (see Bankier, 1988). The parameter can be adjusted according to the importance of the strata estimation and the overall estimation. A value of $\alpha =1$ results in a *proportional to size allocation* of sampling units to the zones/regions of interest and thus favours the overall estimation, while a value of $\alpha =0$ leads to *equal sample sizes* in all zones/regions. A value of $\alpha =0.5$ is used in ALL-EMA, which corresponds to the aim of the survey to produce estimates at both levels of estimation (i.e. zones/regions and total population).

The calibration method described by Deville and Särndal (1992) is then used to determine the indices of interest that satisfy the constraints given in Eqs. (1) and (2). The logistic function is selected from the various calibration functions proposed by Deville and Särndal (1992), as it can impose bounds on the indices and limit the dispersion. Here, the bounds (0.61, 3) are used.

#### Inclusion probabilities

The aim in ALL-EMA is to select a sample of $m=170$ squares with inclusion probabilities proportional to $n_i \, I_i$, where $n_{i}$ approximates the surface area of the accessible agricultural land in BD-Z7 square *i*. The survey should be divided into $r=5$ rotation groups (i.e. panels of the BDM-Z7 survey) $R_1,\dots ,R_t,\dots ,R_T$ of equal size $m/r = 34$. Hence, the selection of sampling units needs to be proportional to $n_i \, I_i$ within each group.

Therefore, the inclusion probabilities in rotation group $R_t$ are described as:

$$\begin{aligned} \pi _{1,i\mid i\in S_0} =\min ( C_t n_i I_i, 1 ), i \in R_t \, \end{aligned}$$

and require that:

$$\begin{aligned} \sum _{i \in R_t} \min ( C_t n_i I_i, 1 ) = \frac{m}{r}=34 \, \end{aligned}$$

The value of $C_t$ was computed with the algorithm given in Tillé (2006, pp. 18–19) using the R package *sampling* (see Tillé & Matei, 2013). This algorithm can assign a probability equal to one to some squares, which means that they are always selected in the sample (Fig. 1 in Supplementary Information). Because of the limited number of BDM-Z7 squares, exact proportionality cannot always be achieved and some deviation from the index of interest $I_i$ prescribed solution has to be accepted.

#### Sampling

A doubly balanced spatial sampling method is used to select the sample of BDM-Z7 squares. The method has been proposed by Grafström and Tillé (2013), and combines the cube method of Deville and Tillé (2004) and the spatial pivotal method of Grafström et al. (2012), which ensures that the sample $S_1$ of squares is spatially spread and balanced for a given vector of auxiliary variables $\textbf{x}_i$, such that:

$$\begin{aligned} \sum _{i=1}^M \textbf{x}_i \approx \sum _{i\in S_1} \frac{\textbf{x}_i}{\pi _{1,i\mid i\in S_0}} \, \end{aligned}$$

In the selection of the first-stage sample $S_{1}$ of BDM-Z7 squares, the following auxiliary variables are used:

$$\begin{aligned} \begin{aligned}&\textbf{x}_i = \left( \pi _{1,i\mid i\in S_0}, \pi _{1,i\mid i\in S_0} U_{1i},\dots ,\pi _{1,i\mid i\in S_0} U_{gi},\dots ,\pi _{1,i\mid i\in S_0} U_{Gi}, \right. \\ {}&\left. \pi _{1,i\mid i\in S_0} V_{1i},\dots ,\pi _{1,i\mid i\in S_0} V_{hi},\dots ,\pi _{1,i\mid i\in S_0} V_{Hi}, n_i , elev_i, 1\right) \,\, \end{aligned} \end{aligned}$$

$elev_i$ is the mean elevation above sea level, measured on the accessible agricultural land in square *i*, and $U_{gi}$ and $V_{hi}$ are indicator variables (i.e. 0/1) for the ERZOs and UZL-HRs.

In order to improve the efficiency of the method, the flight phase of the doubly balanced spatial sampling design is applied separately in each rotation group $R_t$. The method is then reapplied to the rounding problem, as proposed by Chauvet (2009). This procedure provides annual subsamples of equal size that are well spread and almost balanced in each survey year.

The spatial distribution of the final sample selection for ALL-EMA is shown in Fig. 2 of the Supplementary Information, while Figs. 3 and 4 of the Supplementary Information show the corresponding sample proportions in the ERZOs and UZL-HRs.

### Second-stage sample of the baseline habitat plots

The sample of the baseline habitat plots $S_2$ is distributed across the *m* first-stage sample squares by applying grid sampling with a grid spacing of 50 m to a surface area of 0.9025 km$^2$ within each selected BDM-Z7 square. The sample size $n_i$ is thus proportional to the area of the accessible agricultural land $A_i$. The total number of habitat plots in the sample is $n= \sum _{i\in S_1} n_i$, where $n_i\le 361$. With the grid of 361 centre points and plots with a surface area of 10 m^2^, the conditional inclusion probability $\pi _{2,j\mid i\in S_1}$ of the habitat plots is:

$$\begin{aligned} \pi _{2,j\mid i\in S_1} = \frac{10\times 361}{950^2} = 0.004 \,\, \end{aligned}$$

and the total (unconditional) inclusion probability of the habitat plots is:

$$\begin{aligned} \pi _{H,j} = \pi _{1,i} \pi _{2,j\mid i\in S_1}= \pi _{0,i} \pi _{1,i\mid i\in S_0} \pi _{2,j\mid i\in S_1} \,\, \end{aligned}$$

For the structure plots of 200 m$^2$, these probabilities must be multiplied by 20.

### Third-stage sample of the baseline vegetation plots

#### General principles

In the third stage of sampling, unequal probability sampling is again used in combination with balancing and spreading for the selection of an efficient vegetation sample $S_3$. The sample size should be as equal as possible in each BDM-Z7 square to facilitate the organisation of field data collection. Since the squares $S_1$ are selected proportional to $n_i I_i$, the first and third stages of sampling are (almost) self-weighted (for more on this topic see Särndal et al., 1992, pp. 132–154), which reduces the dispersion of the total inclusion probabilities of the vegetation plots.

#### Index of interest

The aim is to give plots located in rarer but biologically important habitat types a better chance of being selected in the sample of vegetation plots. At the same time, it is preferable to avoid collecting redundant information in spatially clumped or species-poor habitat types. Hence, an index of interest $J_{j}$ is defined to guide the sample selection process. The relevant information is obtained from expert opinions on all 91 habitat types and is classified into three levels per variable. The variables refer to different types of interest, as shown in the RMP-biplot in (Fig. 5 of the Supplementary Information). The first axis of the RMP-biplot is used to define the overall weight of the index of interest $J_{j}$.

#### Inclusion probabilities

The sample size is, in principle, fixed to a constant number ($v_0 = 19$) of vegetation plots in each BDM-Z7 square. This number is determined according to a cost model. However, if the number of habitat plots in a BDM-Z7 square is already very small, the sample size may be smaller. The overall size of the sample of vegetation plots is $v = \sum _{i\in S_1} v_i$, with $v_i \le n_i$, the number of vegetation plots in square *i*.

If needed, the sample size is adjusted in the following way:
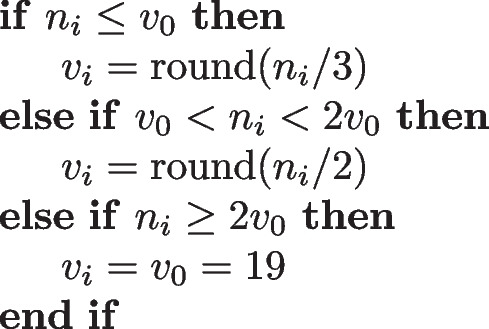


The conditional inclusion probabilities of the vegetation plots in a square can then be written as:

$$\begin{aligned} \pi _{3,j\mid j\in S_2} = \min ( C_{3.i} J_j, 1 ) \, \end{aligned}$$

where the $C_{3.i}$ values need to fulfil the constraints:

$$\begin{aligned} \sum _{j=1}^{n_i} \min ( C_{3.i} J_j, 1 ) = v_i \end{aligned}$$

in each *i*. The $C_{3.i}$ values are again computed with the algorithm described in Tillé (2006, pp. 18–19), and the total (unconditional) inclusion probabilities of the vegetation plots are:

$$\begin{aligned} \pi _{V,j} = \pi _{1,i}\pi _{2,j\mid i\in S_1}\pi _{3,j\mid j\in S_2} \, \end{aligned}$$

#### Sampling

The sample is again drawn according to the doubly balanced spatial sampling design proposed by Grafström and Tillé (2013). The random sample $S_3$ of vegetation plots is balanced on a set of auxiliary variables $\textbf{x}_{V,j}$, such that:

$$\begin{aligned} \sum _{j=1}^{n_i} \textbf{x}_{V,j} \approx \sum _{j\in S_3} \frac{\textbf{x}_{V,j}}{\pi _{3,j\mid j\in S_2}} \, \end{aligned}$$

For the balancing, the variables contained in the following vector are considered:

$$\begin{aligned} \textbf{x}_{V,j} = (\pi _{3,j\mid j\in S_2},1,{elev}_j,{slope}_j, {topo}_j, {east}_j, {north}_j) \end{aligned}$$

where $\pi _{3,j\mid j\in S_2}$ are the prescribed, conditional inclusion probabilities. The remaining variables are derived from a national digital terrain model with a spatial resolution of 25 m. Variables ‘elev’ and ‘slope’ define the elevation above sea level and the slope at the plot location. Variables ‘east’ and ‘north’ are continuous variables indicating the orientation of the plot, calculated as cosine and sine functions of the plot aspect in degrees. Finally, the variable ‘topo’ describes the relative position of a plot along a topographic gradient ranging from exposed ridge, hilltop, shoulder, back-slope and foot-slope to flat plain, sink and distinct ditch. All these variables are expected to affect the spatial distribution of habitats and species in the landscape.

### Sampling design of the EFA survey

The baseline survey is complemented by a dynamic survey of the temporary EFA polygons within the same selection of BDM-Z7 squares. Sampling is carried out in the survey year using the latest EFA polygons (i.e. from the previous year) in an updated sampling frame and has to be repeated for each survey run. The main objective of this sampling is to cover as many EFA types as possible within a square and nationwide. In addition, the sampling should again evenly distribute the workload across the sampling squares and ensure a geographically spread and balanced sample within the squares. To achieve this, sampling procedures similar to those used in the baseline survey are applied. The details are described in Section 1 of the Supplementary Information.

## Estimators

### Estimation of a plot mean and area share

For the estimation of a mean at the plot level, the Hájek estimator can be used (Hájek et al., 1971). Suppose that $y_j$ is the measurement of variable *y* in plot *j* for the habitat sample $S_2$, then the estimator is:

3 $$\begin{aligned} \widehat{\overline{Y}}_H= \frac{1}{\sum _{j\in S_2} \frac{1}{\pi _{H,j}}}\sum _{j\in S_2} \frac{y_j}{\pi _{H,j}} \, \end{aligned}$$

If the measurement $y_j$ of variable *y* is made in plot *j* for the vegetation sample $S_3$, the estimator is:

$$\begin{aligned} \widehat{\overline{Y}}_V= \frac{1}{\sum _{j\in S_3} \frac{1}{\pi _{V,j}}}7 \sum _{j\in S_3} \frac{y_j}{\pi _{V,j}} \, \end{aligned}$$

Accordingly, the estimator of a mean of the EFA sample $S_{3_E}$ is:

$$\begin{aligned} \widehat{\overline{Y}}_{HV_E}= \frac{1}{\sum _{j\in S_{3_E}} {\frac{1}{\pi _{HV_E,j}}}}\sum _{j\in S_{3_E}} {\frac{y_j}{\pi _{HV_E,j}} } \end{aligned}$$

Applying the estimators of a mean value to data on the presence or absence of a category makes it possible to estimate the area share (proportion) of that category.

### Variance in the ALL-EMA survey

The full sampling design of the baseline survey has as many as four stages ($S_0$, $S_1$, $S_2$, $S_3$). Accordingly, the EFA sample also includes up to four stages ($S_{0_E}$, $S_{1_E}$, $S_{2_E}$, $S_{3_E}$). In the four-stage case, the variance should have four terms, which is difficult to handle in practice.

The first-stage sample $S_1$ is not strictly a second-stage sample of $S_0$, but a second-phase sample in the sense of the terminology used by Särndal et al. (1992, pp. 133–150 and 343–368). Indeed, there are no primary and secondary units for passing from $S_0$ to $S_1$ because they are both samples of squares. Sample $S_1$ is just a subset of $S_0$. Analogously, $S_3$ is just a subset of $S_2$ without any intermediate units. The only intermediate units are the squares. This also applies in the EFA sample. The variance estimation of ALL-EMA is therefore simplified by the assumption that the sampling design only consists of two stages: the selection of the squares and the selection of the circular plots.

For the BDM-Z7 sample, the selection of squares $S_0$ is systematic. However, the variance is usually estimated under the assumption that the squares are selected with simple random sampling (Plattner et al., 2004; Lanz, 2013; BDM Coordination Office, 2011; BDM Coordination Office, 2014). This estimator probably slightly overestimates the variance. Indeed, if a spatial correlation exists, systematic sampling provides generally better estimates than simple random sampling.

In contrast, to further simplify the estimation of variance in the more complex ALL-EMA design, it is assumed that $S_1$ is selected from the entire agricultural area of Switzerland *A*, with replacement and unequal probabilities according to the Hansen-Hurwitz scheme (Hansen & Hurwitz, 1943) with drawing probabilities $p_i=\pi _{1,i}/m$. With this assumption, the calculation of the variance is still laborious. However, when this complex variance needs to be estimated, the variance estimator simplifies dramatically. While the variance is written as the sum of as many terms as there are degrees in the design, the simplified variance estimator consists of only one term (see Särndal et al., 1992; Chauvet & Vallée, 2020; Tillé, 2020, pp. 158–161). One only has to calculate a simple sum of squares to estimate the variance. In this way, all stages of the sampling design are well considered.

The Hansen-Hurwitz assumption applies both to the BDM-Z7 sample of squares $S_0$ and to the sample $S_1$ of $S_0$. On the one hand, this approach overestimates the variance by a factor with an order of magnitude equal to the inverse of correction for the finite population: $(M_0-1)/(M_0-m)$, where $M_0$ approximates the number of squares in the total area *A*. In our case, with $m=170$ and $M_0 \approx 15,688$, the factor is equal to $(15688-1)/(15688-170) = 1.010891$, which is very small. On the other hand, the Hansen-Hurwitz assumption also neglects the effect of the systematic selection of $S_0$ and the balancing design of $S_1$. The variance is thus additionally slightly overestimated. In any case, it is impossible to estimate the variance in an unbiased way in systematic sampling because some joint inclusion probabilities are null. The assumption applied here thus provides confidence intervals that are slightly conservative but easy to calculate.

Specifically, the estimator of the variance for a plot mean simplifies to:

4 $$\begin{aligned} \widehat{var}(\widehat{\overline{Y}}) = \frac{1}{\widehat{N}^2}\frac{1}{m(m-1)}\sum _{i\in S_1}\left( \frac{\widehat{Y}_i}{p_i}-\widehat{Y}\right) ^2 \end{aligned}$$

where $\widehat{N}$ is an estimator of the total area *A* divided by the plot area size. $\widehat{Y}_i$ is the estimated total in square *i*, and $\widehat{Y}$ is computed as follows:

$$\begin{aligned} \widehat{Y} = \sum _{j\in S_1} \frac{\widehat{Y}_i}{\pi _{1,i}} \end{aligned}$$

Estimator Eq. (4) can be used for both the baseline samples and the additional EFA sample. $\widehat{N}$ and $\widehat{Y}_i$ are, however, estimated differently. For the baseline habitat type sample:

$$\begin{aligned} \widehat{Y}_i =\sum _{j\in Q_i \cap S_2} \frac{y_j}{\pi _{2,j\mid i\in S_1}} \end{aligned}$$

For the baseline vegetation sample:

$$\begin{aligned} \widehat{Y}_i =\sum _{j\in Q_i \cap S_3} \frac{y_j}{\pi _{2,j\mid i\in S_1}\pi _{3,j\mid j\in S_2}} \end{aligned}$$

For the EFA sample:

$$\begin{aligned} \widehat{Y}_i =\sum _{j\in Q_i \cap S_E} \frac{y_j}{\pi _{2_E,j\mid i\in S_{1_E}} \pi _{3_E,j\mid j\in S_{2_E}}} \end{aligned}$$

where $Q_i$ denotes the set of plots in square *i*. Cases of spatial mismatch between a EFA polygon and a plot area can be considered by computing:

$$\begin{aligned} \widehat{Y}_i = \sum _{j\in Q_i \cap S_E} \frac{y_j w_j}{\pi _{2_E,j\mid i\in S_{1_E}} \pi _{3_E,j\mid j\in S_{2_E}}} \end{aligned}$$

where $w_j$ is again the proportion of the plot area located within the EFA polygon. Similarly, $\widehat{N}$ in Eq. (4) can be defined according to the sampling design as:

$$\begin{aligned} \widehat{N}_H =\sum _{j\in S_{2}} \frac{1}{\pi _{H,j}},\widehat{N}_{V} =\sum _{j\in S_{3}} \frac{1}{\pi _{V,j}},\;or\; \widehat{N}_{HV_E}= \sum _{j\in S_{3_E}} {\frac{w_j}{\pi _{HV_E,j}}} \end{aligned}$$

## Sample efficiency assessment

Planning comprehensive biodiversity monitoring of biodiversity on agricultural land at a large scale requires complex sampling design decisions. In the case of ALL-EMA, this process was supported by examining the estimation efficiency of different sampling designs using Monte Carlo simulations and power analyses based on modelled habitat data. Here, we therefore present results from the baseline habitat sample $S_2$ and also use real data to check the validity of the habitat model. However, the principles of this assessment also apply to the baseline vegetation sample $S_3$ and the additional EFA samples, as their sampling replicates the sampling techniques of the sample of squares $S_1$ within those squares. The structure of this section then follows the natural stages of the survey planning phase. After decisions on sampling procedures have been made, the issue of sample size and distribution is addressed, and finally, the changes that the habitat survey can be expected to detect are assessed.

### Methods

#### Artificial population of habitat types

Assessing the estimation efficiency of the habitat sample $S_1$ required knowledge on the spatial distribution of the habitat types in the agricultural land of Switzerland. Due to the lack of real data in the planning phase, an artificial population of habitat types was created in the initial BDM-Z7 grid sample of squares $S_0$. To do this, the sample size of a habitat type *l* in a square $n_{i,l}$ was modelled by means of a double Poisson model with $n_{i,l}= 1/2 \times Pois(1,2) \times Pois(p_{l,g},n_{i})$, where $n_{i}$ is the number of habitat grid plots falling within the agricultural land of a square $A_i$ and $p_{l,g}$ is the expected proportion of habitat type *l* in the corresponding ERZO *g*. The models were run independently for each habitat type, so that the total sample size within a square differed randomly from the intended sample size $n_{i}$. The values of $n_{i}$ were known from the database, whereas the values of $p_{l,g}$ were based on expert knowledge about the occurrence probability of a habitat type within an ERZO. As the expert knowledge was incomplete, only 67 habitat types with assigned occurrences were modelled.

In this way, the abundance of habitats at the level of squares (i.e. raster points within the squares) was modelled. In doing so, the uneven distribution of habitat types between the ERZOs was mimicked, and the amount of agricultural land in the squares was considered. By choosing a double Poisson model, considerable variation between the squares was also added. However, an even distribution of the habitats within the ERZOs was assumed, as no information about this was available in the planning phase.

#### Effect of stratified balancing

A Monte Carlo simulation study was performed, using the initial BDM-Z7 squares $S_0$ with modelled habitats from the ‘‘Artificial population of habitat types’’, to investigate the benefit of a stratified balanced square sample $S_1$ for the baseline habitat sample $S_2$ compared with a pure unequal probability sampling design. Testing the effect of spatial spreading additional to stratified balancing was not useful, due to the lack of spatial structure within the strata in the habitat model. Therefore, 300 draws of the first-stage unequal probability sampling of ALL-EMA were simulated with and without balancing on $\textbf{x}_i$, and the proportional surface area occupied by the 67 target habitat types in the agricultural land of Switzerland was repeatedly estimated using Eq. (1) in the Supplementary Information. The coefficient of variation was then calculated as a percentage across sample draws to express the accuracy of the proportion estimates for each design. To better see the effect of sampling, the proportion estimates of the habitat types were repeatedly summed into 17 broader habitat classes, corresponding to the second level of detail (i.e. habitat group) in the habitat typology of Delarze et al. (2015), and their coefficient of variation was calculated again across sample draws.

#### Effect of sample clustering

Budget constraints limit the number of sampling units that can be observed in the field. Reducing the number of sampling squares reduces travel costs and allows a higher sampling rate within the squares. However, plots that are close to each other tend to be similar, which has a negative impact on the statistical efficiency of the sample. To assess the effect of sample clustering on the plot-level estimation of habitat types in ALL-EMA, an additional Monte Carlo simulation study was performed based on the habitat distribution model described in the ‘‘Artificial population of habitat types’’ section and a global cost model. The cost model assumed a fixed cost per habitat record and travel time (i.e. the time to travel to the squares and the time to travel between plots within a square), which allowed comparison of cluster versions with equal costs. Here, the Monte Carlo simulation compared square sample sizes *m* ranging from 100 to 300. Survey costs were kept equal by adjusting the affordable sample size $n_{i}$ to the selected square sample size and modelling an artificial habitat population per cluster version using the adjusted sample size $n_{i}$. Subsequently, 300 draws of the ALL-EMA sample of squares $S_1$ were performed per cluster version and habitat model. To quantify the accuracy of each cluster version, the proportions of the habitat types were again estimated per sample draw using Eq. (1) in the Supplementary Information, the individual estimates were summed within the broader habitat classes, and the coefficient of variation was calculated as a percentage across sample draws.

#### Power analyses for change detection

Power analyses were performed to examine the changes that are likely to be detected by the baseline habitat sample $S_{2}$. The analyses considered two-stage sampling with unequal probabilities and estimated the minimum number of squares required to detect relevant changes in the surface area of a habitat type by using the equation for permanent (re-measured) sampling units:

$$\begin{aligned} m_{min} \ge \frac{s^2(2-2\rho )(Z_{1-\alpha /2}+Z_{1-\beta })}{(\delta {\overline{Y}})^2} \,\, \end{aligned}$$

where $\overline{Y}$ denotes the current proportion (mean) of a habitat type and $s^2$ is the variance of the respective square-level proportions. $\rho$ is the correlation between paired square proportions, and $\delta$ is the rate of change to be detected at a significance level of $\alpha$ and a power of $1 - \beta$. $Z_{p}$ is the constant exceeded with probability *p* by a standard normal $\mathcal {N} (0; 1)$ random variable. In this study, the aim was to detect relevant changes $\delta$ of 10%, 20%, and 30% at a confidence level of 68% ($\alpha = 0.32$) and a power of $1 - \beta = 0.5$, thus corresponding to the detection of ‘significant standard errors’. This low threshold is a useful reference value for early change detection in the establishment phase of long-term monitoring programmes (Mahrer & Vollenweider, 1983). Three classes (0.8, 0.9, 0.95) were assumed for the correlation between paired square proportions $\rho$. The correlation depends on the fluctuation of a habitat type over time and was expected to increase with the time span between re-measurements in a way similar to the rate of change $\delta$.

Power analyses were performed in the planning phase using the modelled habitats from the ‘‘Artificial population of habitat types’’ section. The results can be found in Section 5 of the Supplementary Information. Here, existing survey data were used to present an up-to-date picture. A first set of power analyses used the observations from the first survey run of ALL-EMA from 2015 to 2019 to estimate $\widehat{\overline{Y}}$ and $\widehat{s^2}$ for each habitat type. In doing so, $\widehat{\overline{Y}}$ was calculated using Eq. (3) from the ‘‘Estimators’’ section. To estimate $\widehat{s^2}$, the adjusted Hansen-Hurwitz estimator defined in Section 4.3 of the Supplementary Information was used to calculate $\widehat{var}(\widehat{\overline{Y}}_1)$, the variance of a global mean square proportion. $\widehat{s^2}$ was then calculated as:

$$\begin{aligned} \widehat{s{^2}} = {\widehat{var}(\widehat{\overline{Y}}_1)} {m} \end{aligned}$$

Next, available replicate data from 68 squares observed in 2020 and 2021 were used to perform an additional power analysis, with $\widehat{\delta }$ and $\widehat{\rho }$ also estimated from real data, this time distinguishing between three power classes $1 - \beta$. The *wCorr* package (Bailey & Emad, 2021) in R was used for the calculation of $\widehat{\rho }$, allowing the inclusion of survey weights. This gave the actual number of squares needed to confirm the changes observed so far (after 5 years) with a confidence level of 68%.

**Fig. 4:**
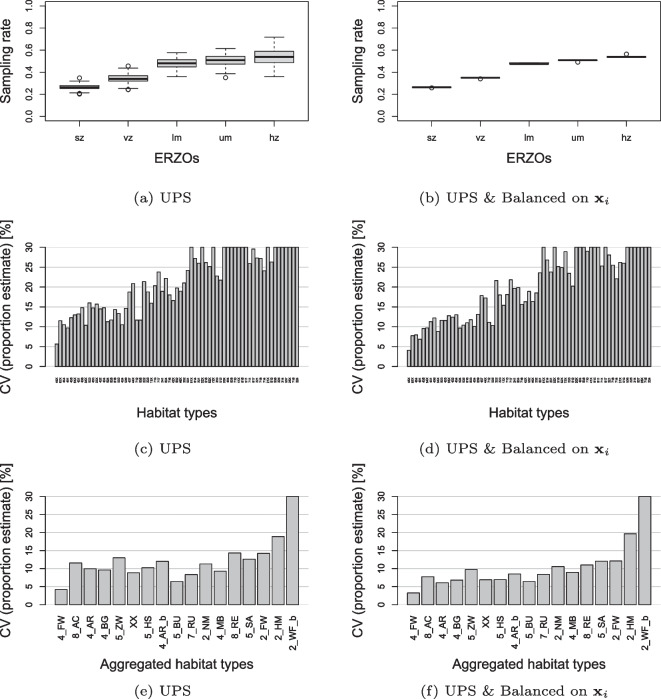
Monte Carlo simulations exploring the effect of stratified balancing in ALL-EMA first-stage unequal probability sampling (UPS) of squares on sampling rates in ERZOs **a**, **b**, and national estimation accuracy of 67 target habitat types **c**, **d** and 17 aggregation classes **e**, **f**. The boxplots show sampling rates as proportions within strata (ERZOs), and the bar graphs show estimation accuracy as coefficient of variation (CV). The categories are displayed in decreasing order of (expected) nationwide frequency. On the left side **a**, **c**, **e**, the sampling design is unbalanced. On the right-hand side **b**, **d**, **f**, the sampling design is balanced on $\textbf{x}_i$. The graphs show the results of 300 draws of the sampling designs from the initial grid of BDM-Z7 squares $S_0$ with an artificial population of 67 habitat types. The ERZOs are abbreviated as follows: sz, summering zone; vz, valley zone; lm, lower mountain zones I and II; um, upper mountain zones III and IV; hz, hill zone

**Fig. 5:**
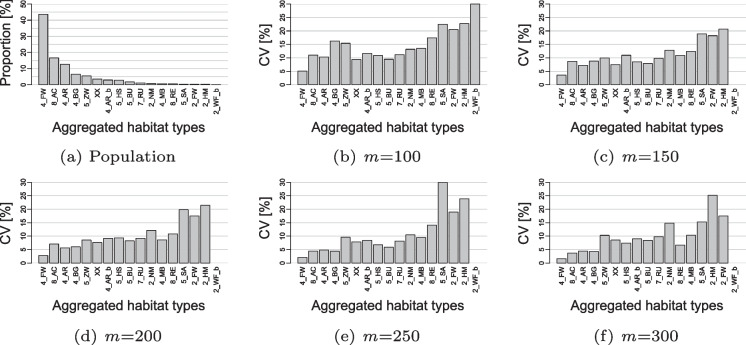
Monte Carlo simulations exploring the effect of cost-neutral sample spreading (decreased clustering of the habitat sample) for estimating the national proportion of 17 aggregated habitat types, where type 2_WF_b occurs only in cluster version **b**. Bar graphs **b** to **f** show the precision of the proportion estimates in terms of coefficient of variation (CV). The simulations are based on 300 draws of the ALL-EMA square sample $S_1$ with varying sample size *m* from the grid of BDM-Z7 squares $S_0$ with a modelled population of 67 habitat types for each scenario of *m*. **a** shows, for reference, the distribution of aggregated habitat types in the modelled population of scenario **b**

### Results and discussion

#### Effect of stratified balancing

**Fig. 6:**
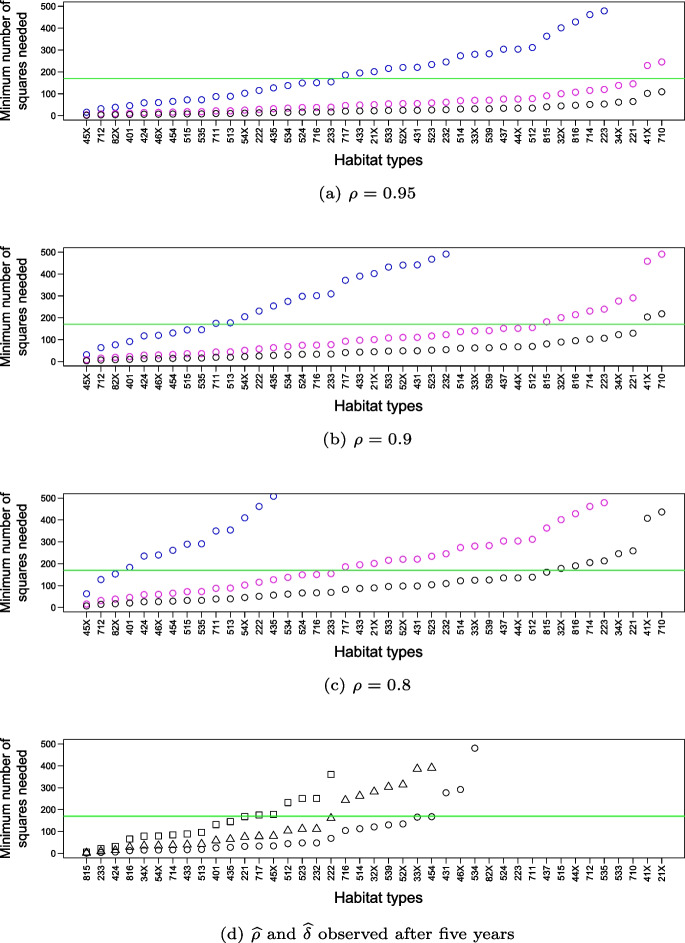
National change detection for the target habitat types as a function of relevant changes **a**, **b**, **c** and actual changes observed after 5 years **d**. Circles indicate the minimum number of sample squares *m* needed to confirm a certain degree of habitat change at a confidence level of 68% and a power of $1 - \beta = 0.5$. In **a**, **b**, and **c**, correlation values of $\rho = 0.95$, $\rho = 0.9$, and $\rho = 0.8$ are assumed for the changes to be detected. The different colours represent three classes of change $\delta$ (blue: 10%, pink: 20%, green: 30%). In contrast, **d** shows the minimum number of squares needed to detect actual changes $\widehat{\delta }$ with actual correlation values $\widehat{\rho }$ observed after 5 years. The different symbols represent the three power classes (square: 0.9, triangle: 0.7, circle: 0.5). In each panel, the symbols under the green line at $m=170$ indicate the changes that can be detected with the square sample size of ALL-EMA. The graphs include all 41 habitat types that were recorded in at least 10 of the 68 revisited squares. Missing symbols are outside the displayed range

The proportion estimates of the modelled habitat types were clearly better with stratified balancing than with pure unequal probability sampling in the Monte Carlo simulations (Fig. 6). The increase in accuracy was 14.5% on average (i.e. mean relative reduction in the coefficient of variation) and was particularly evident for the frequent habitat types on the left side of Fig. 4d and f. The improvement occurred even though the modelled habitat population is a grid sample $S_0$ and thus already well distributed over the ERZOs. However, simulations that also included geographic spreading in the sample design (not presented here) showed no further improvement, which was not surprising since the artificial population was constructed without spatial structure in the ERZOs.

Balancing and geographic spreading is assumed to be most effective in the third stage of ALL-EMA sampling. The spatial structure is expected to be highest within the squares. In addition, the variables used for balancing in this stage of sampling are likely to have an even greater impact on the indicators and target items.

#### Effect of sample clustering

As expected, the estimation quality of 17 aggregated habitat types increased with larger square sample sizes in the Monte Carlo simulations (Fig. 5). The increase in estimation accuracy (without 2_WF_b) was substantial from $m=100$ to $m=150$ (19.8% on average), while the improvements were rather small and not even consistent for the larger square sample sizes (25.6% for $m=200$, 23.3% for $m=250$, and 28.7% for $m=300$ compared with $m=100$). Based on this result, strong clustering with 170 squares was ultimately selected, as it favoured inference at the square (i.e. landscape) level, a predefined secondary objective in the ALL-EMA survey.

#### Power analyses for change detection

The power analyses based on data from the first survey run (Fig. 6a, b and d) show similar statistical power to that expected from the simulations before field work (see Fig. 6 in the Supplementary Information). The small but systematic underestimation of statistical power with the simulations suggests that the double Poisson habitat model was a fairly rigorous study population. The additional power analysis, which considered actual patterns of change (Fig. 6d), shows that, after repeating all 170 squares, ‘significant standard errors’ can be expected to be detected with a power of 0.5 for the majority (i.e. 25 out of 41) of the habitat types studied. Indeed, the rate of change $\widehat{\delta }$ (median = 0.17) already observed after 5 years corresponds to the relevant range studied. The correlation values $\widehat{\rho }$ (median = 0.86) were also in the middle of the investigated range.

## Conclusions

This study shows how nationwide monitoring of habitats, structures, and plants can be carried out to efficiently capture the complex patterns of biodiversity on agricultural land at all relevant scales. The sampling design presented combines several techniques for this purpose:

- The samples are clustered into a selection of BDM-Z7 squares to reduce travel time and to facilitate both plot (i.e. local) and landscape-level estimation.
- The squares and plots are selected with inclusion probabilities proportional to predefined weights (i.e. index of interest). Thus, the samples are directed towards the small ERZOs and UZL-HRs (square sample), the less common but relevant habitat types (baseline vegetation sample), and a large number of EFA types (EFA sample).
- Using two-phase sampling allows for a more comprehensive coverage of plant species, despite the lack of *a priori* information about them.
- The selection of squares is temporally stratified to enable a fixed sample size per survey year and to match a predefined annual rotation plan.
- Applying the methods of spatially balanced sampling and doubly balanced sampling ensures that the samples of all stages and years are well spread in the geographic space.
- These methods also ensure that the samples and their annual parts are balanced across regions and important environmental gradients, i.e. the Horvitz-Thompson estimator of the total of the balancing variables is almost equal to the population total. The sample variance of the balancing variables can thus be minimised.
- Using self-weighting enables fixed sample sizes $v_i$ and $e_i$ at the third stage with minimal dispersion of inclusion probabilities.
- The dynamic sampling of the EFAs takes into account the temporary nature of agri-environmental measures.

These properties ensure that the estimators are very efficient in extrapolating the results of the sample to subpopulations (in particular the predefined target groups) or in total (i.e. the entire agricultural land of Switzerland). Sampling with unequal probabilities equals stratified sampling with unequal sampling rates but allows a general rather than a stratified evaluation. The (annual) sample size within regions and squares is still controlled by sampling with fixed sample sizes, stratified balancing, and self-weighting, which is convenient for the organisation of field work. Additional balancing on environmental gradients is efficient if the indicator to be estimated correlates with these gradients, while geographic spreading of the samples avoids the collection of redundant information from nearby sites. Both features exceed the capabilities of stratified approaches. Extending the advanced sampling design to dynamic sampling of agri-environmental measures is particularly important to evaluate the allocation of subsidies to promote biodiversity-friendly practices.

A Monte Carlo simulation study using modelled habitat data showed the positive effect of stratified balancing for the baseline habitat sample. The average reduction of the sample variance was 14%. Another simulation examined how to balance the workload between sampling squares to find an optimal trade-off between plot-level and landscape-level estimation. This analysis indicated relatively small improvements in habitat estimates with square sample sizes larger than 150. Power analyses with real data showed that after a full survey run, changes could be detected for 25 habitat types with a confidence level of 68% (i.e. ‘significant standard error’) and a power of 0.5. These results are similar to those of power analyses with modelled habitat data, confirming the double Poisson habitat model as a suitable study population in the planning phase of the monitoring programme.

The approach presented here offers several advantages that have not yet been exploited in operational biodiversity monitoring on complex agricultural land. In this way, the approach could be a useful example for improving future monitoring networks and for obtaining meaningful and efficient samples for operational use, including at the local scale. To provide guidance on how to implement the advanced sampling techniques, an annotated R script, which demonstrates the first stage selection of squares of ALL-EMA, is provided additional to this article.

### Supplementary Information

Below is the link to the electronic supplementary material.

Supplementary file1 (PDF 1.22 MB)

## Data Availability

An annotated R script presenting the first-stage sampling of squares is provided, with input data, in the EnviDat repository: https://doi.org/10.16904/envidat.402.
