# Supplementary material for: Integrating spatial and ecological information into comprehensive biodiversity monitoring on agricultural land
Source: Environ Monit Assess. 2023 Sep 7;195(10):1161. doi: 10.1007/s10661-023-11618-7 (PMC10485118; doi:10.1007/s10661-023-11618-7)
Supplement: Supplementary file 1 — Supplementary file1 (PDF 1.22 MB) [file 10661_2023_11618_MOESM1_ESM.pdf]

Integrating spatial and ecological information into comprehensive biodiversity monitoring on agricultural land

Klaus Thomas Ecker[1*], Eliane Seraina Meier[2] and Yves Tillé[3]

[1*]Biodiversity and Conservation Biology Research, Swiss Federal Institute for Forest, Snow and Landscape Research WSL, Birmensdorf, Switzerland.
[2]Agroecology and Environment, Agroscope, Zurich, Switzerland.
[3]Institute of Statistics, University of Neuchâtel, Neuchâtel, Switzerland.

*Corresponding author(s). E-mail(s): klaus.ecker@wsl.ch;

Supplementary Information

1 Full sampling design of the EFA survey

1.1 Inclusion probabilities for the three stages of EFA sampling

In contrast to the baseline survey, the temporary ecological focus areas (EFAs) are assessed in the full square kilometre of the first-stage sample of BDM-Z7 squares S_{1_E}. Hence, the inclusion probabilities of the initial BDM-Z7 squares in the EFA survey are $\pi_{0_E,i} = 1/48 = 0.02083333$ in the densified grid of the Southern Alps and the Jura region and $\pi_{0_E,i} = 1/96 = 0.01041667$ otherwise (see Figure 1 in the article), and the conditional inclusion probabilities of the first-stage sampling squares are identical to those of the baseline survey (see Section 4.2.3 in the article):

$$\pi_{1_E,i|i\in S_{0_E}} = \pi_{1,i|i\in S_0}$$

In the second stage, one random plot centre is selected within each EFA polygon, resulting second-stage sampling plots that may extend the border of the EFA polygon. In fact, some EFA categories are very small or are linear, causing an inevitable spatial mismatch for the given plot sizes of 10 and 200 m^2. The ratio between the actual size of the plot in the polygon and the size of the polygon therefore defines the conditional second-stage inclusion probability of these plots:

$$\pi_{2_E,j|i\in S_{1_E}} = a/A_{P,j|i}$$

where a is the actual size of a plot j and $A_{P,j|i}$ is the area of the corresponding EFA polygon.

The number of third-stage EFA plots should be fixed within the squares. The sizes are defined, according to a cost model, to be fixed at $e_0 = 14$ in the EFA survey. However, since only one plot is selected per polygon, the sample size e_i is capped at the number of EFA polygons p_i occurring within a square i, so that $e_i = \min(e_0,\ p_i)$ and the total sample size is $e = \sum_{i\in S_{1_E}} e_i$, with $e_i \leq p_i$.

In addition to enabling fixed sample sizes, the inclusion probabilities have to satisfy two opposing conditions. They have to be proportional to the area of a polygon $A_{P,j|i}$, but at the same time promote uniform sampling rates of EFA categories regardless of their size. The opposing goals are achieved by first defining target sample sizes per category and then calculating the inclusion probabilities individually for each EFA category. The procedure is as follows. First, the predefined sample size e_i is split among the EFA categories occurring in a square to obtain individual target sample sizes that are as uniform as possible. This involves hierarchically ranking the categories occurring in a square according to predefined national abundance classes and then their local frequency in these classes. The units of sample size e_i are then distributed in

turn among the individual categories, beginning with the rarest one. Categories that are used up are skipped in the turn-wise procedure. Thus, surplus units are assigned to the rarest categories that are not yet consumed. The resulting partition of the sample size e_i is used to compute the prescribed inclusion probabilities separately for each EFA category. Here, the EFA categories are denoted by $E_1, \ldots, E_k, \ldots, E_K$.

The inclusion probabilities in EFA category E_k can be written as:

$$\pi_{3_E,j|j \in S_{2_E}} = \min(C_k A_{P,j|i}, 1), j \in E_k$$

and C_k must be determined under the constraint:

$$\sum_{j \in E_k} \min(C_k A_{P,j|i}, 1) = e_k$$

where e_k is the individual target sample size of category E_k.

The value C_k is again defined by the algorithm described in Tillé (2006, pp. 18–19). The total (unconditional) inclusion probability of a EFA plot is then given by:

$$\pi_{HV_E,j} = \pi_{1_E,i} \pi_{2_E,j|i \in S_{1_E}} \pi_{3_E,j|j \in S_{2_E}}$$

1.2 Three stages of EFA sampling

The first stage of EFA sampling corresponds to the selection of BDM-Z7 squares in the baseline sampling design. In the second stage, a random plot is located in each EFA polygon of a selected square. In the third stage, a sample of these plots is selected with inclusion probabilities $\pi_{3_E,j|j \in S_{2_E}}$. The third-stage sample is again balanced and spread using the method of Grafström and Tillé (2013). The auxiliary variables considered for the balancing are:

$$\mathbf{x}_{E,j} = (elev_j, F_{1j}, \ldots, F_{rj}, \ldots, F_{Rj}, A_{P,j})$$

Variable $elev_j$ again denotes the elevation above sea level of the plot centre. $A_{P,j}$ is the area of the corresponding EFA polygon. The variable F_{rj} contains the indicator variables of a coarse classification of the EFA categories $F_{1j}, \ldots, F_{rj}, \ldots, F_{5j}$ describing five categories of increasing land use intensity: 1 = extensively used meadows of high biological quality, 2 = pastures and meadows of medium use intensity but high biological quality, 3 = areas with trees or hedgerows, 4 = remaining grassland not including categories 1 and 2, and 5 = fields, vineyards and special cultures.

The selected sample of plots S_{3_E} is balanced on the variables $\mathbf{x}_{E,j}$, such that:

$$\sum_{j=1}^{e_i} \mathbf{x}_{E,j} \approx \sum_{j \in S_{3_E} \cap \text{Square } i} \frac{\mathbf{x}_{E,j}}{\pi_{3_E,j|j \in S_{2_E}}}$$

Thus, the sampling variance is reduced among the land-use intensity categories and across elevation and polygon size. The sample is also spread in geographic space.

2 Temporal organisation of the survey

The temporal organisation of the survey is set to follow the rotational plan of the BDM-Z7 survey. This plan splits the BDM-Z7 squares into five groups, which are surveyed in five successive years. The groups are aligned with diagonal lines in the grid sample and are hence well distributed. To reproduce the five rotational groups, the temporal groups are used as strata in the first-stage sampling to obtain a balanced sample of equal size for each year. Thus, the temporal distribution of the square sample contains one-fifth of the square sample for each year of the survey. The annual subsets are consistent with BDM-Z7 rotations.

The baseline survey repeatedly records structures, neophytes, habitats and vegetation in fixed plots within the squares. However, the static design of the permanent plots is not appropriate for surveying EFAs over time, as the contracts for their management have time limits. The minimum duration to receive subsidies is eight years, i.e. just less than the time span of two survey periods. To comply with the dynamic character of EFAs, an independent vegetation sample must be drawn from the currently valid EFA area for each survey run. The power loss arising from surveying unpaired plots has to be compensated by higher sample rates.

In repeated survey sampling, a panel is optimal to estimate evolutions and to minimise the variation between sites, whereas a complete rotation is better for accurate transversal estimates. During monitoring, however, with a panel only the information about temporal variation increases and that about spatial variation remains the same. Temporal shifts in the target universe or individual target groups may even cause bias with a full panel (Gruijter et al., 2006).

The given BDM-Z7 system of cyclic rotation with periodic updates after five years is thus a compromise for estimating both the evolutions and the transversal parameters. For the baseline survey, there is a complete rotation of non-overlapping annual samples between waves 1 and 2, 2 and 3, 3 and 4, and 4 and 5. Thus, the evaluations cannot be accurately estimated from one year to the next. Nevertheless, waves 1 and 6 have the same statistical units. Years 1, 2, 3, 4 and 5 can also be compared with years 6, 7, 8, 9 and 10. Thus, the sampling design is optimal to accurately estimate five-year evolutions.

This sampling interval should be appropriate for measuring the changes of interest, apart from cyclic annual variation. Rapid annual change is not expected with plant species. Annual fluctuations due to climate are likely to exceed longer-term changes. Recording species composition consistently is difficult and values can vary greatly among observers (Vittoz et al., 2010). Further variation is introduced by the timing of management and the phenology of the species (Vymazalova et al., 2012). It is not possible to fully control both

in a national survey. Resulting annual variations in the data are smoothed by estimating mid-term evolutions. However, responses of plant species assemblages to change are known to be time-lagged due to underlying population processes. If there is rapid change, the estimates from paired observations are rather conservative in time. Constructing statistics on state parameters from aggregated annual waves is a time-lagged approximation.

In addition to the statistical advantages, the annual rotation system simplifies the organisation of the field campaign, as field work can be evenly distributed over the full survey period. This is important for the successful recruitment, continuous participation, and training of the field team. It ensures that the expertise of the field team is at a constant high level and minimises observer variance and bias.

3 Characteristics of the final sample

The final samples of ALL-EMA have some important characteristics. The first-stage sample of squares S_1 is selected with unequal probabilities from the initial grid of BDM-Z7 squares. The distribution of these inclusion probabilities is shown in Figure 1. The geographic distribution of the final selection of 170 squares S_1 is shown in Figure 2. As intended, the final square sample S_1 favours the smaller agricultural production zones (ERZOs) and biogeographic regions (UZL-HRs) (Figure 3 and 4). The baseline vegetation sample S_3 is again selected with unequal probabilities from the baseline habitat sample S_2. The weights (i.e. index of interest) used to determine the inclusion probabilities of the habitat plots S_2 within a square are shown in Figure 5.

4 Estimators part 2

Simple point and variance estimators for plot-level estimation are presented in Section 5 of the article. Here, other important estimators are presented.

4.1 Estimation of a global square mean

The estimation of a global square mean is of particular interest for estimating indicators at the landscape scale, and involves computing intermediate square estimates using the Hájek estimator (Hájek, 1971). If $\widehat{\overline{Y}}_i$ is the intermediate estimate for a mean of variable y in square i, as calculated from the plot measurements y_j within a square i using the equations given in the article (Section 5.2), then the overall mean $\widehat{\overline{Y}}_1$ of the squares is given by:

$$\widehat{\overline{Y}}_1 = \frac{1}{\sum_{i \in S_1} \frac{1}{\pi_{1,i}}} \sum_{i \in S_1} \frac{\widehat{\overline{Y}}_i}{\pi_{1,i}} \tag{1}$$

For the EFA sample, S_1 and $\pi_{1,i}$ have to be replaced by S_{1_E} and $\pi_{1_E,i}$, respectively.

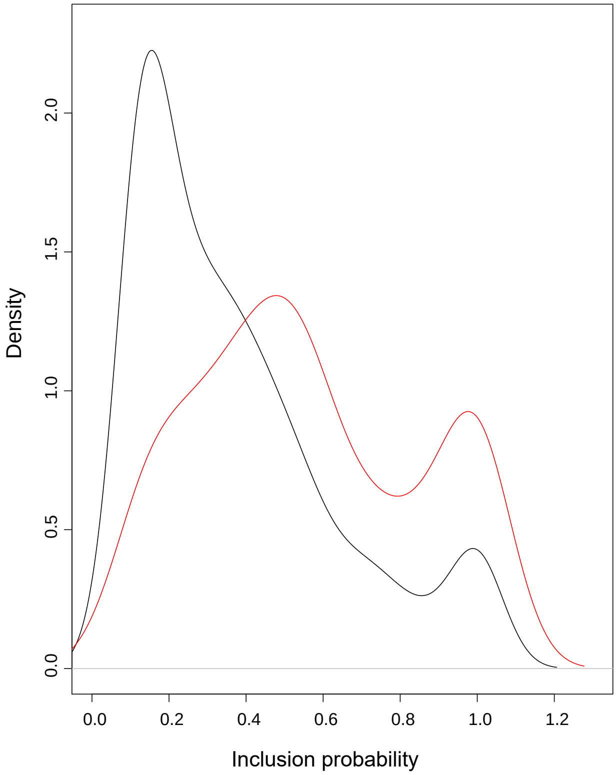

Fig. 1: Density distribution of the inclusion probabilities of the 455 BDM-Z7 squares in the sample frame (black line) and of the final sample of 170 selected squares (red line). The maximum value of 1 is assigned to 30 squares after additionally assigning maximum sampling weights (i.e. index of interest) to the squares with $n_i > 50$ in Canton Valais and Ticino (for the sake of simplicity not not described in the article). These squares will definitely be selected in the sample. The bandwidth of the smoothing function in the kernel estimator is 0.03928. The positive density of inclusion probabilities less than zero and greater than one are artefacts of the smoothing method

4.2 Extrapolation to the totals

To estimate the totals from the baseline samples, the ratio estimator is used here, and the Hájek estimator of a mean is simply multiplied by the target area A and divided by the plot size a:

$$\widehat{Y} = \widehat{\overline{Y}} \frac{A}{a}$$

Similarly, for estimating totals from the EFA samples, the following equation is used:

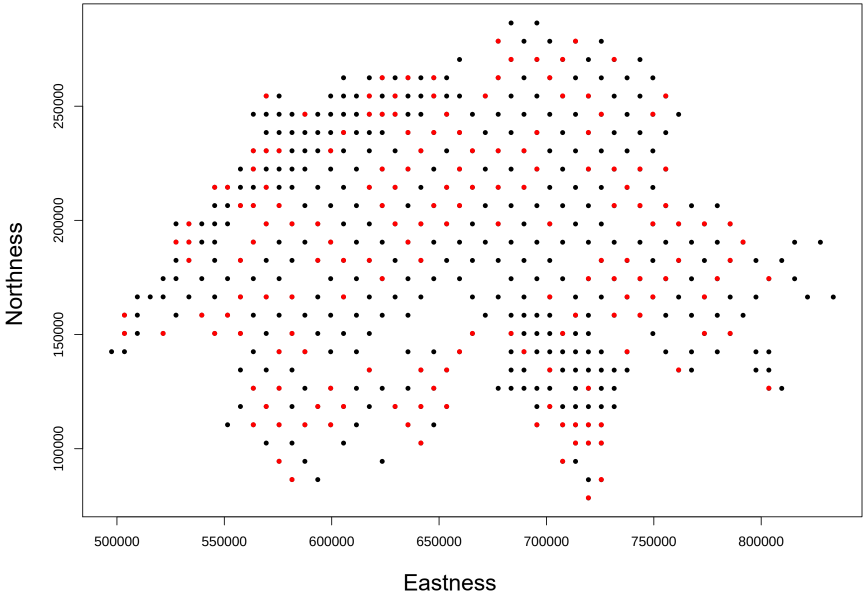

Fig. 2: Final selection of a spread and balanced sample of 170 squares (red dots) from the sample frame (black dots: squares not selected from the sample frame). The sampling intensity is increased in the small target regions, i.e. Southern Alps and Jura mountains

$$\widehat{Y}_E = \widehat{\overline{Y_E}} \frac{A_E}{a}$$

However, the target areas A and A_E are not known from the database and have to be estimated. $A_{CH} = 41{,}285$ km^2 is the area of Switzerland. From this population, the part of the territory that lies outside the scope of the BDM-Z7 sample $S0$, such as terrain containing glaciers, lakes and other inaccessible areas, first has to be excluded to define the target area A_0. As there is no nationwide database of polygons describing the sampling area of the BDM-Z7 survey, A_0 has to be reproduced from the initial BDM-Z7 square sample S_0 by estimating:

$$\widehat{A}_0 = A_{CH} \frac{\sum_{i \in G_{CH}} \mathbb{1}[i \text{ is in the scope of } S_0]}{\sum_{i \in G_{CH}} 1}$$

where G_{CH} is a grid representation of Switzerland with a spacing of one kilometre between grid points. $\mathbb{1}[i$ in the scope$]$ equals 1 if the square is within the scope of the BDM-Z7 sample S_0 and 0 if not. In a selected square of S_1, it is then possible to estimate the part of the square that is in the scope of the ALL-EMA samples for habitats (S_2) and vegetation (S_3) using:

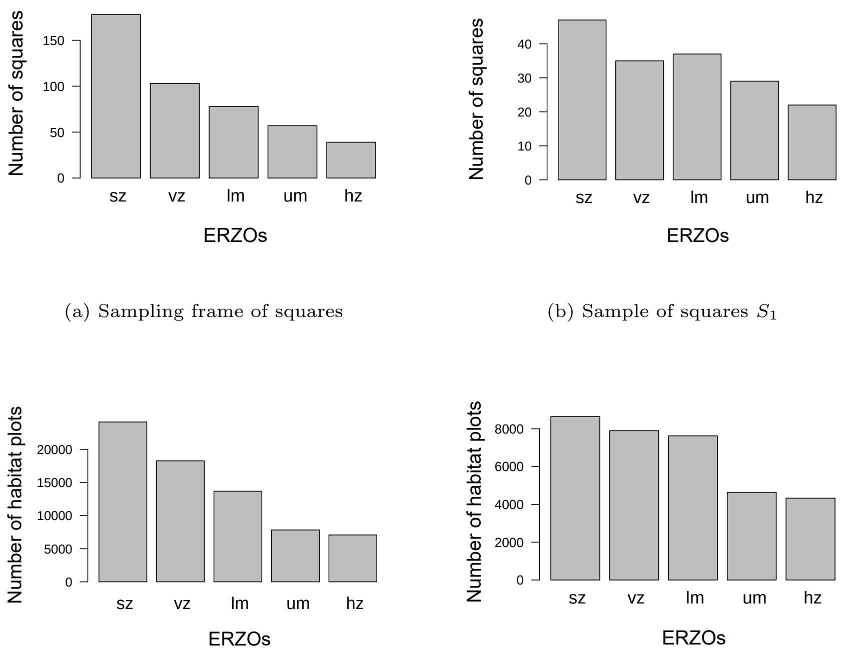

(a) Sampling frame of squares

(b) Sample of squares S_1

(c) Sampling frame of baseline habitat plots

(d) Sample of baseline habitat plots S_2

Fig. 3: Disproportional representation of the agricultural production zones (ERZOs) (sz = summering zone, vz = valley zone, lm = lower mountain zones I and II, um = upper mountain zones III and IV, hz = hill zone) in the final sample compared with the sampling frame. The first row shows the frequency of the squares. The squares are assigned to the zones where the majority of their plots n_i belong. The second row shows the frequency of the baseline habitat plots, as indicated by the preliminary delineation of the target area

$$G_i = \frac{n_{f,i}}{361}$$

where $n_{f,i}$ is the final number of plots in square i confirmed in the field to be within the scope of ALL-EMA. The maximum number of habitat plots in a square is 361. The estimator of the area A within the scope of ALL-EMA is then:

$$\widehat{A} = \widehat{A}_0 \frac{\sum_{i \in S_1} \frac{G_i}{\pi_{1,i}}}{\sum_{i \in S_1} \frac{1}{\pi_{1,i}}}$$

Accordingly, the target area of the EFA sample is only known for the selected squares. Thus, the total area can be computed as:

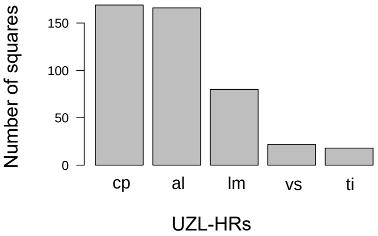

(a) Sampling frame of squares

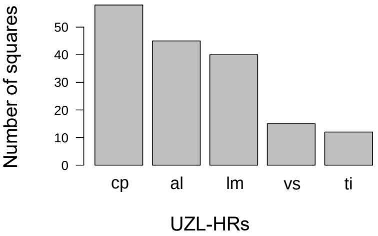

(b) Sample of squares S_1

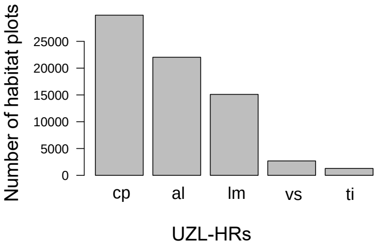

(c) Sampling frame of baseline habitat plots

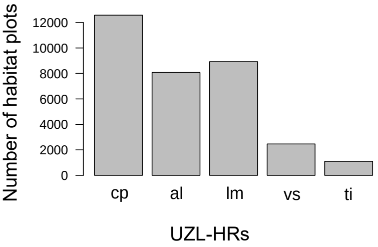

(d) Sample of baseline habitat plots S_2

Fig. 4: Disproportional representation of the biogeographic regions (UZL-HRs) (al = Alps, cp = Central Plateau, lm = lower mountains, vs = canton of Valais, ti = canton of Ticino) in the final sample compared with the sampling frame. The first row shows the frequency of the squares. The squares are assigned to the regions where the majority of their plots n_i belong. The second row shows the frequency of the baseline habitat plots, as indicated by the preliminary delineation of the target area

$$H_i = \frac{A_{E,i}}{1000^2}$$

where $A_{E,i}$ is the area of EFA polygons in square i and 1000^2 is the total area of a square in the EFA survey. The total EFA area is then estimated as:

$$\widehat{A}_E = \widehat{A}_0 \frac{\sum_{i \in S_1} \frac{H_i}{\pi_{1_{E,i}}}}{\sum_{i \in S_1} \frac{1}{\pi_{1_{E,i}}}}$$

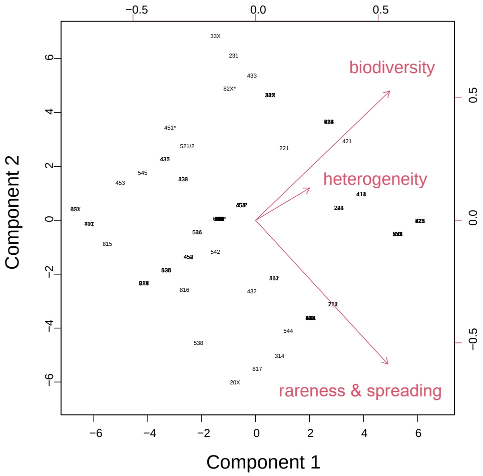

(a) Distribution of 91 habitat types along predefined sampling interests

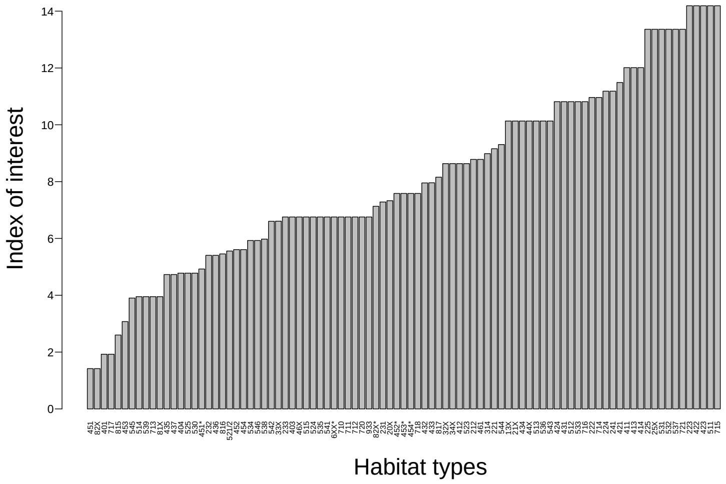

(b) Overall sampling interest of 91 habitat types

Fig. 5: Definition of the sampling interest of 91 habitat types in the baseline vegetation sample. The position of the habitat types across three different sampling interests (i.e. importance for biodiversity, heterogeneity of the species composition, rareness and spreading) is presented in a biplot. The overall sampling interest is obtained from the first axis of the biplot. The values are assigned to the baseline habitat plots in a square which gives the index of interest J_j. This raises the sample rates of the less frequent but spatially dispersed, biologically important and species-rich habitat types

4.3 Variance estimation of a global square mean

The estimator given by equation 4 in the article can be further adapted to estimate the variance of a global square mean $\widehat{var}(\widehat{\overline{Y}}_1)$. $\widehat{Y}_i$ is replaced by the individual square estimates $\widehat{\overline{Y}}_i$, and $\widehat{Y}$ is computed as:

$$\widehat{Y} = \sum_{i \in S_1} \frac{\widehat{\overline{Y}}_i}{\pi_{1,i}} \; or \; \widehat{Y}_E = \sum_{i \in S_{1_E}} \frac{\widehat{\overline{Y}}_i}{\pi_{1_E,i}}$$

$\widehat{N}$ estimates the total number of squares in the target population:

$$\widehat{N}_1 = \sum_{i \in S_1} \frac{1}{\pi_{1,i}} \; or \; \widehat{N}_{1_E} = \sum_{i \in S_{1_E}} \frac{1}{\pi_{1_E,i}}$$

The variance estimator for the plot-level and square-level means can be extrapolated to an estimator of the variance for the respective totals:

$$\widehat{var}(\widehat{Y}) = \widehat{var}(\widehat{\overline{Y}}) \left(\frac{\widehat{A}}{a}\right)^2 \; or \; \widehat{var}(\widehat{Y}_E) = \widehat{var}(\widehat{\overline{Y}}_E) \left(\frac{\widehat{A}}{a}\right)^2$$

where $\widehat{A}$ is the estimated target area of a sample, and a is the area of the plot or square unit to be estimated.

4.4 Estimation in space and time

Statistical inference in space and time has to consider both the spatial and the temporal sampling pattern. An overview of inference techniques for standard equal probability monitoring designs which involve sampling in space and time is given by Gruijter et al. (2006). According to their classification, the present survey design uses a five-period synchronous sampling pattern (see Gruijter et al., 2006, Figure 14.6, p. 216) with systematic sampling in time but complex unequal probability sampling in space. The methods they describe in chapter 15 for inferring global quantities of state, change and evolution can be adapted to the present sampling design simply by using the spatial variance estimator of the present spatial design as a plug-in.

Accordingly, state quantities may be inferred from single annual waves by using the spatial variance estimator. Mid-term state (i.e. spatio-temporal) quantities can be estimated in the same way from the aggregated waves of a survey period. Similarly, change may be inferred from non-overlapping waves or paired observations. Short-term change has to be estimated from non-overlapping annual waves by computing the differences in the annual state estimates (e.g. means). The variance is the sum of the respective annual variance estimates (see Gruijter et al., 2006, Equation 15.18, p. 228). Since the non-overlapping samples do not contain the same units, the non-overlapping waves have a small negative correlation (Qualité and Tillé, 2008), but this cannot be estimated and can simply be neglected. The waves are thus treated as if they are independent. To estimate mid-term change, the paired observations

(aggregated or not) can be used. Since the spatial means of the two sampling dates are correlated in the paired samples, the equation of the summed variance (see Gruijter et al., 2006, equ. 15.20, p. 229) includes a covariance term, which reduces the variance estimate. Hence, it is more efficient to estimate mid-term change from paired observations than from independent samples. In classical testing, the equation is equivalent to the common t-test for paired observations. However, the covariance term is difficult to compute in our complex spatial design, and it is simpler to compute the difference between the paired observations (aggregated or not) at time t and $t-5$. The variance is then simply estimated by applying the variance estimator of the spatial design to the differences, as is usual when estimating change with paired data. The principle of the t-test for paired data can thus be applied, although the variance estimator must take the specificity of the sampling design into account. Change in totals or spatial fractions can be estimated in the same way as change in spatial means.

To estimate evolution (i.e. the spatial mean temporal trend), different methods are used for paired or unpaired sampling patterns (see Gruijter et al., 2006, p. 230). Accordingly, mid-term evolution within a survey period may be assessed from the non-overlapping annual waves by first calculating the state parameters (i.e. point and variance estimate) from each sampling time. A model can then be fitted on the annual point estimates to infer the trend parameter β and its variance. The model is based on weighted least squares fitting, with weights inversely proportional to the variances of the individual point estimates.

To estimate evolution from paired sampling patterns, Gruijter et al. (2006, p. 231) propose estimating a model parameter at each sampling location, and in a second step inferring its spatial mean using the spatial estimators of the design. We believe, however, that the coefficient β should be estimated, similar to the unpaired situation, from annual estimates except that the covariances for the lag of five must be taken into account. More specifically, for a long enough period (more than five years), an effect should be added to the model for the paired data, as in the analysis of longitudinal data, using a non-diagonal covariance matrix for the point estimate, with correlations between t and $t-5$, $t+1$, and $t-4$, and so on. This procedure also takes into account spatial autocorrelation.

5 Power analyses for change detection using simulations

Determining the minimum number of sampling squares needed to capture area changes in the target habitat types requires knowledge of the spatial and temporal variation of these target variables. However, only rough knowledge of their regional distribution was available from experts in the planning phase of ALL-EMA. The simulations described in Section 7.1 of the article are thus used to obtain a rough picture of the changes that could be captured by the

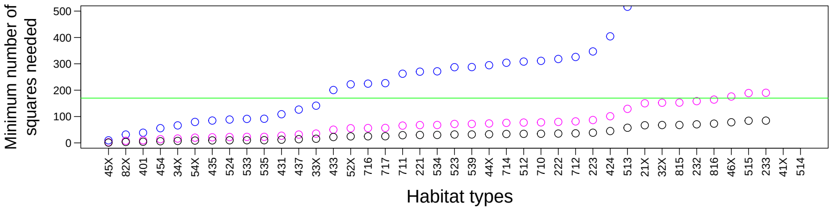

(a) $\rho = 0.95$

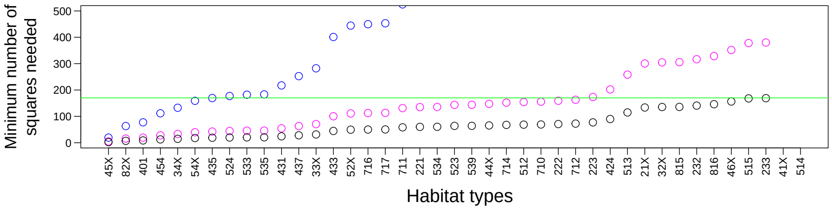

(b) $\rho = 0.9$

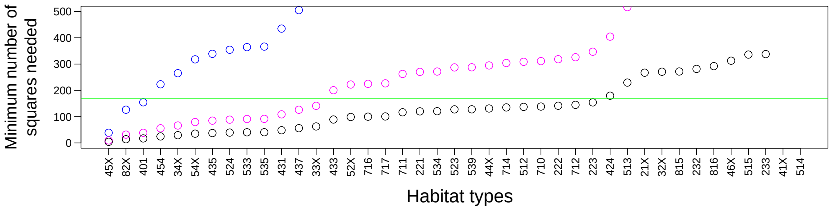

(c) $\rho = 0.8$

Fig. 6: National change detection for the target habitat types as a function of magnitude and correlation of change. The graphs are based on simulations of the ALL-EMA sampling design and assume correlation values of (a) $\rho = 0.95$, (b) $\rho = 0.9$, and (c) $\rho = 0.8$ between paired square estimates. In each case, the circles indicate the minimum number of squares m needed to confirm a certain degree of habitat change with a power of $1 - \beta = 0.5$ and a confidence level of 68%. The colours represent three classes of change (blue: 10%, pink: 20%, green: 30%). The circles under the green line at $m = 170$ thus indicate the changes that should be detected by ALL-EMA in the planning phase

ALL-EMA sampling design (Figure 6). To this end, $\widehat{\overline{Y}}$ and $\widehat{s^2}$ are estimated as described in Section 7.3 of the article for each sample draw, and average values are derived for use in Equation 5 of the article.

References

Grafström, A. & Tillé, Y. (2013). Doubly balanced spatial sampling with spreading and restitution of auxiliary totals. *Environmetrics*, 14(2):120–131.

Gruijter, J. d., Bierkens, M., Brus, D., & Knotters, M. (2006). *Sampling for Natural Resource Monitoring*. Springer, Berlin Heidelberg New York.

Hájek, J. (1971). Discussion of an essay on the logical foundations of survey sampling, part on by D. Basu. In Godambe, V. P. & Sprott, D. A., editors, *Foundations of Statistical Inference*, p. 326, Toronto, Canada. Holt, Rinehart, Winston.

Qualité, L. & Tillé, Y. (2008). Variance estimation of changes in repeated surveys and its application to the Swiss survey of value added. *Survey Methodology*, 34:173–181.

Tillé, Y. (2006). *Sampling Algorithms*. Springer, New York.

Vittoz, P., Bayfield, N., Brooker, R., Elston, D. A., Duff, E. I., Theurillat, J.-P., & Guisan, A. (2010). Reproducibility of species lists, visual cover estimates and frequency methods for recording high-mountain vegetation. *Journal of Vegetation Science*, 21(6):1035–1047.

Vymazalova, M., Axmanova, I., & Tichy, L. (2012). Effect of intra-seasonal variability on vegetation data. *Journal of Vegetation Science*, 23(5):978–984.
